# Gaussian Approach for the Synthesis of Periodic and Aperiodic Antenna Arrays: Method Review and Design Guidelines

**DOI:** 10.3390/s21072343

**Published:** 2021-03-27

**Authors:** Giulia Buttazzoni, Fulvio Babich, Stefano Pastore, Francesca Vatta, Massimiliano Comisso

**Affiliations:** Department of Engineering and Architecture, University of Trieste, Via A. Valerio 10, 34127 Trieste, Italy; babich@units.it (F.B.); pastore@units.it (S.P.); vatta@units.it (F.V.); mcomisso@units.it (M.C.)

**Keywords:** antenna arrays, Gaussian approach, excitation synthesis, position synthesis, power pattern synthesis, 5G systems

## Abstract

This paper presents a complete overview of the recently developed Gaussian approach for the synthesis of both periodic and aperiodic linear antenna arrays in conjunction with an exhaustive numerical investigation of the achievable performance. The position and excitation synthesis problems are jointly modeled to enable a direct mutual comparison between the two strategies. To this aim, different parameter settings are selected to analyze the results in terms of achieved beamwidth and maximum sidelobe levels as a function of the array aperture and of the number of radiating elements. The insights inferred from this numerical investigation are exploited to derive a novel first-step procedure with the purpose of enabling an antenna engineer to quickly identify the most suitable design approach, thus reducing the time required for antenna system development.

## 1. Introduction

The diffusion of the fifth generation (5G) communication system is significantly growing, with many experimental testbeds already available all over the world [1,2]. The 5G technology, with the objectives of connectivity for billion devices worldwide, of zero latency, and of highly increased data rates, is expected not only to revolutionize the cellular communications, by partly incorporating Gigabit WiFi networks and enabling machine-to-machine as well as vehicle-to-vehicle connectivity, but also to have a dramatic impact on many other disciplines, such as medicine and industry [3,4,5,6]. Of course, significant technological improvements in the antenna array design field are mandatory to support such challenging expectations.

Therefore, despite its remote origin [7], the antenna array synthesis problem remains of considerable importance in the 5G scenario [8], and many antenna proposals have been conceived in the recent years for addressing different issues of the millimeter-wave (mmWave) environment. In particular, in [9], a radiating structure was proposed for supporting the dedicated short range links arising in vehicular communications. A linear antenna array (LAA) composed of eight open-slot planar inverted-F antennas (PIFAs) for 5G mobile terminals was presented in [10]. Additionally, in [11], an eight-element phased array for 5G mobile communications was developed for operations in the 28 GHz band requiring high spatial coverage. A versatile technique was designed in [12], where the authors proposed an algorithm simultaneously enabling the synthesis of the far-field pattern, the selection of the polarization, the reduction of near-field amplitude, and the control of the dynamic range ratio (DRR) of the excitations. An antenna array for 5G cellular systems was developed in [13] by adopting a planar structure consisting of four PIFA pairs placed on the corners of a mainboard. In [14], four four-element LAAs were designed and manufactured by employing substrate-integrated waveguides intended for usage in 5G mobile devices. Different types of planar arrays operating at mmWave frequencies and suitable for mobile handsets were investigated in [15], whereas, in [16,17], 4×4 and 8×8 planar arrays were respectively proposed for 5G base stations. Of course, when the frequency is high and/or the array elements are closely packed, mutual coupling phenomena arise, which should be taken into account. Importantly, the works in [18,19,20,21,22,23,24,25,26,27,28,29] proposed a number of solutions to control the mutual coupling for different technologies, including microstrip patches and substrate integrated waveguides (SIW), and for different applications, including multiple input multiple output (MIMO) systems. In [30], the authors presented an algorithm for the synthesis of random geometries of virtual sensor antenna arrays with maximum mainlobe and minimum sidelobe levels (SLLs) as well as null control. The proposed approach is based on the particle swarm optimization (PSO) algorithm. In [31], a synthesis method was developed for automotive radar applications at 24 GHz. The algorithm is based on the concept of a virtual antenna array and on the method of moments/genetic algorithm (MoM/GA). In the context of LAAs, the recently developed Gaussian method represents a very useful design tool. It was originally proposed for the position synthesis (PS) of aperiodic linear arrays with uniform amplitude distribution of the element excitations [32]. Subsequently, rectangular geometries [33] and scanning applications [34] have also been successfully considered for the PS problems. Finally, it has been extended to include the excitation synthesis (ES) of linear arrays with fixed element positions [35,36], which are still considered a valid candidate for supporting the communications in the emerging 5G technology [37,38,39]. In both the PS and ES cases, the algorithm has provided a very good performance, also in comparison with other existing synthesis techniques.

According to this background, this paper focuses on LAAs by firstly presenting a comprehensive description of the Gaussian approach for both periodic and aperiodic structures, with the aim of providing an overview of the applicability contexts and of the potentialities of the method. This description is provided by jointly modeling the PS and ES cases to enable a direct comparison between the two approaches. Subsequently, to deepen the above listed considerations, a detailed numerical investigation is presented by analyzing the achievable performance in terms of beamwidth (BW) and maximum SLL as a function of the array length; of the number of elements; and more importantly, of the linear array type. A given number of elements offers to the designer, in fact, the same number of degrees of freedom in both a periodic and an aperiodic array, but the influence on the results is considerably different. To the best of authors’ knowledge, this relevant aspect has not been previously analyzed. Moving from these considerations, a first-step procedure enabling the antenna array designer to preliminarily identify the better approach is developed with the aim of significantly reducing the number of required simulations and thus the realization time.

The remainder of this paper is organized as follows. Section 2 formulates the problem and introduces the Gaussian approach. Section 3 discusses the numerical investigation. Section 4 presents the parametric analysis and the design procedure. Finally, Section 5 summarizes the most relevant conclusions.

## 2. The Problem and the Gaussian Approach

With reference to a Cartesian system O(x,y,z), consider the array factor of a LAA composed by *N* identical elements located on the *z*-axis at the positions $z_{n}$ (n=1,…,N). According to [7], this array factor can be expressed as follows:

(1) $$F\left(a,z;\theta \right)=\sum_{n=1}^{N}a_{n}\exp\left(jkz_{n}\cos\theta \right),$$

where $a=[a_{1},\cdots ,a_{N}]$ is the vector of the complex excitations, $z=[z_{1},\cdots ,z_{N}]$ is the vector of the element positions, *j* is the imaginary unit, and k=2π/λ is the wavenumber, with λ being the wavelength. As usual, θ is the co-latitude or azimuth angle (i.e., the angle measured from the *z*-axis). The development of the Gaussian approach for the synthesis of pencil beams generated by (1) originates from two main observations.

**Figure 1:**
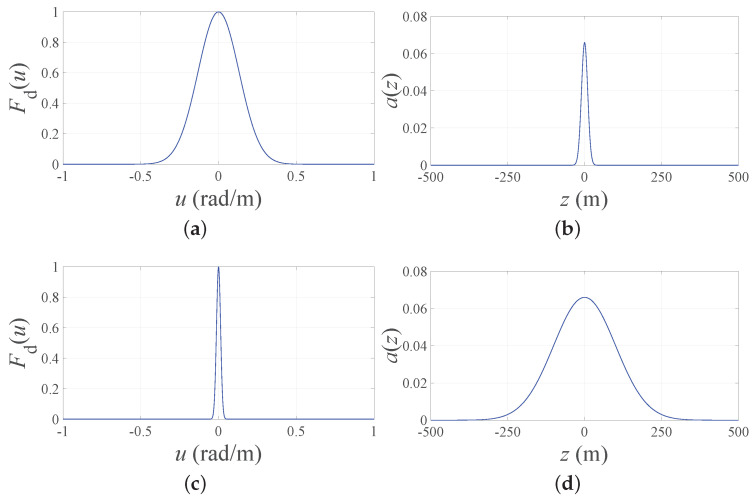
(**a**) $F_{d}\left(u\right)$ obtained by setting BW = 1${}^{\circ }$ and b = 3 in (3). (**b**) Corresponding continuous source a(z). (**c**) $F_{d}\left(u\right)$ obtained by setting BW = 0.1${}^{\circ }$ and b = 3 in (3). (**d**) Corresponding continuous source a(z). As it may be noticed from each pair of figures, the narrower the desired pattern, the wider the continuous source.

- First, a Gaussian function may be used to approximate a pencil beam with a desired BW by suitably controlling its standard deviation. More precisely, by denoting with u=kcosθ, the desired Gaussian function:
  (2) $$F_{d}\left(u\right)=\exp\left(-\frac{u^{2}}{2\sigma^{2}}\right),$$
  has a unitary maximum amplitude for θ=π/2 (u=0) and a–b dB amplitude for θ=(π±BW)/2 (u=kcos[(π±BW)/2]) if its standard deviation σ is as follows [32]:
  (3) $$\sigma =k\sqrt{\frac{10}{b\ln10}}\cos\left(\frac{\pi +\mathrm{BW}}{2}\right).$$
- Second, a Fourier transform relation holds between a continuous linear infinite source a(z) and its far-field pattern [40]. Moreover, the Fourier transform of a Gaussian function may be evaluated as another Gaussian function with reciprocal standard deviation (Figure 1).
  Hence, if $F_{d}\left(u\right)$ in (2) represents the desired far-field pattern, the corresponding continuous source is immediately evaluated as follows:
  (4) $$a\left(z\right)=\frac{1}{2\pi }\int_{-\infty }^{\infty }F_{d}\left(u\right)\exp\left(-jzu\right)du=\frac{\sigma }{\sqrt{2\pi }}\exp\left(-\frac{\sigma^{2}z^{2}}{2}\right).$$
  The expression for the continuous infinite source a(z) in (4) that exactly produces the pattern in (2) can be successively truncated to a finite length *L* and processed in order to approximate the array factor in (1). Thanks to the Gaussian nature of a(z), closed-form expressions are obtained in both the PS and the ES problems.

These two aspects constitute the key points of the Gaussian approach and determine its effectiveness. Before describing in detail the Gaussian approach, it is worth noting that, if the PS of aperiodic LAAs with uniform amplitude distribution of the excitations is considered, $a_{n}=a_{0},\;\forall n$ and z represents the unknown vector of the positions. Conversely, when the ES of periodic arrays is considered, $z_{n}=nd,\;\forall n$ (with *d* being the constant inter-element distance) and a represents the unknown vector of the excitations. However, although the degrees of freedom of the problem are *N* in both cases, the optimization variables in (1) appear in the exponential terms when the PS is addressed and as coefficients when the ES is addressed instead. Thus, they are expected to have a different influence on the optimization results.

### 2.1. Position Synthesis—Aperiodic Arrays

**Figure 2:**
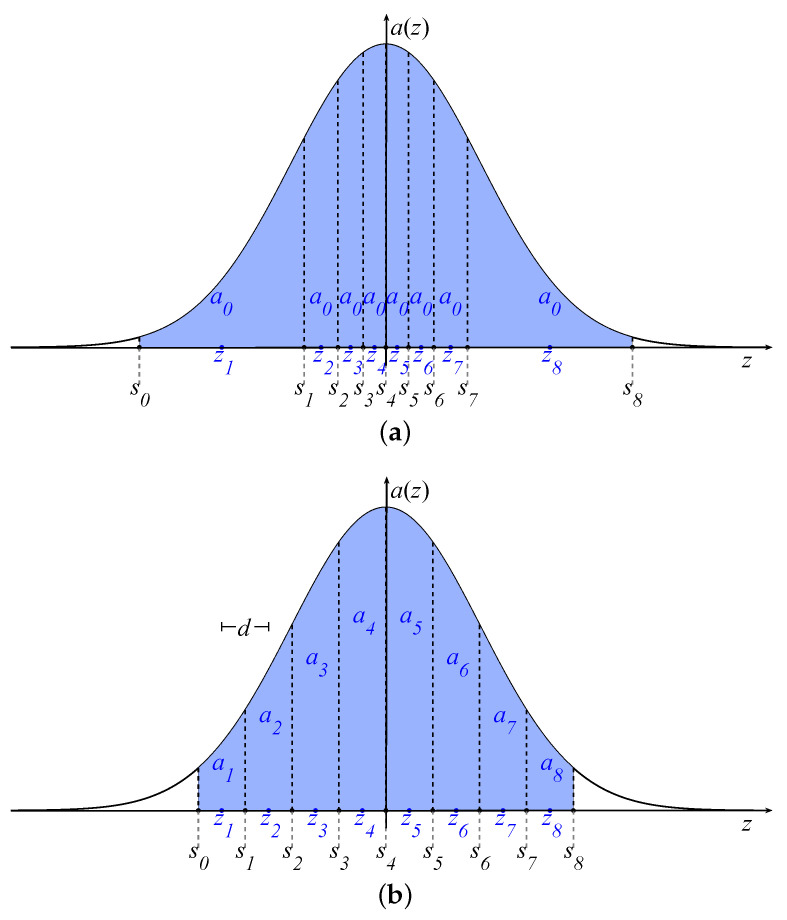
(**a**) Position synthesis (PS): the interval extrema $s_{n}$ are evaluated by imposing the equi-area requirement of the density tapering approach. The final positions $z_{n}$ are the middle points of each interval. (**b**) Excitation synthesis (ES): each element excitation $a_{n}$ is evaluated as the area between the *z*-axis and the graph of a(z) in the interval $\left[z_{n}-d/2,z_{n}+d/2\right]$.

Linear arrays having all the elements with the same excitation are attractive in many applications in which only the positions are optimized [41,42,43,44,45,46,47]. Exactly this scenario represents the so-called PS problem. For this problem, the Gaussian approach developed in [32] adopts a density tapering technique [48], which is graphically depicted in Figure 2a. According to this technique, the area between the *z*-axis and the graph of the function a(z) is subdivided into *N* intervals with the same area:

(5) $$a_{0}=\frac{1}{N}\int_{-L/2}^{L/2}a\left(z\right)dz=\frac{1}{N}\;\mathrm{erf}\left(\frac{\sigma L}{2\sqrt{2}}\right),$$

where *L* denotes the array aperture and

(6) $$\mathrm{erf}\left(x\right)=\frac{2}{\sqrt{\pi }}\int_{0}^{x}\exp\left(-t^{2}\right)dt,$$

identifies the error function. The extrema of these intervals are determined by imposing

(7) $$\int_{-L/2}^{s_{n}}a\left(z\right)dz=na_{0},$$

which, thanks to the Gaussian expression of a(z), has a closed-form solution:

(8) $$s_{n}=\frac{\sqrt{2}}{\sigma }\;{\mathrm{erf}}^{-1}\left[\left(\frac{2n}{N}-1\right)\mathrm{erf}\left(\frac{\sigma L}{2\sqrt{2}}\right)\right],$$

with ${\mathrm{erf}}^{-1}\left(x\right)$ being the inverse error function. Once the extrema $s_{n}$ are computed by (8), the optimal positions can be chosen in the middle points of the intervals as follows:

(9) $$z_{n}=\frac{s_{n-1}+s_{n}}{2},\;n=1,\cdots ,N.$$

Alternatively, the optimal positions could be chosen at the barycenters of the intervals [45], even if, with the Gaussian approach, the two choices essentially give the same results [32]. To conclude, the Gaussian approach for the PS of linear aperiodic arrays radiating pencil beams can be summarized as follows. For a source with a given length *L* and consisting of a determined number of elements *N*, first, approximate the desired pencil beam using a Gaussian function through the specification of its standard deviation σ; then use (8) to evaluate $s_{n}$; and finally use (9) to obtain the element positions, $z_{n}$.

### 2.2. Excitation Synthesis—Periodic Arrays

The position synthesis with uniform amplitude distribution is interesting in many applications. However, it is also well known that the limited degrees of freedom may not provide some desired radiation characteristics. Indeed, amplitude tapering of the excitations allows one to better exploit the degrees of freedom of the problem when the uniform amplitude is not a mandatory requirement. Therefore, the Gaussian approach has been also efficiently used for the ES of periodic linear arrays [35]. For this second possibility, consider the continuous source in (4) and *N* periodic positions $z_{n}=nd$. According to these settings, the element excitations can be chosen as follows:

(10) $$a_{n}=\frac{1}{2}\left\{\mathrm{erf}\left[\frac{\sigma }{\sqrt{2}}\left(z_{n}+\frac{d}{2}\right)\right]-\mathrm{erf}\left[\frac{\sigma }{\sqrt{2}}\left(z_{n}-\frac{d}{2}\right)\right]\right\},$$

which identifies the area between the *z*-axis and the graph of the function a(z) in each interval. This procedure is graphically depicted in Figure 2b, which gives an immediate association between the areas and the element amplitudes. From this figure, it can also be immediately argued that a narrow source distribution a(z) inherently produces, using (10), an excitation vector with high DRR, which is defined as follows:

(11) $$\mathrm{DRR}\left(a\right)=\max|a_{n}|/\min|a_{n}|.$$

On the contrary, a flat source distribution inherently results in an excitation vector with a low DRR. To conclude, the Gaussian approach for the ES of linear periodic arrays radiating pencil beams can be summarized as follows. For a source with a given length *L* and consisting of a determined number of elements *N* with a constant interelement distance *d*, first, approximate the desired pencil beam using a Gaussian function through the specification of its standard deviation σ and then use (10) to evaluate the element excitations $a_{n}$. A detailed application example is illustrated in Section 4, which provides the reader with a comprehensive overview of the method.

## 3. Numerical Investigation

In [32,35], the performance of the Gaussian approach for the PS and ES of broadside patterns has been compared with existing methods. A summary of this comparison is reported in Table 1 and Table 2, respectively. In particular, the Gaussian approach for PS has been compared in [32] with both stochastic and deterministic state-of-the-art methods [41,43,46,49,50], revealing a better performance in terms of maximum SLL in almost all considered cases (Table 1). In [35], the Gaussian approach for ES has been compared with the classical Dolph–Chebishev method, which optimizes the relation between BW and maximum SLL. As it can be seen from Table 2, the Gaussian approach provides satisfactory results. Of course, the obtained BW can be wider or the maximum SLL can be higher, but in both examined cases, two significant benefits may be observed. First, the DRR of the excitations is lower. Second, the percentage of the power radiated in the sidelobe region is lower.

Moving from this preliminary assessment of the capabilities that characterize the Gaussian approach, the aim of this study is to relate the design parameters to the algorithm performance to provide to the antenna engineer a useful tool for preliminary design choices. To this purpose, in the sequel of this section, novel scenarios are examined for both uniform amplitude aperiodic arrays and periodic arrays with amplitude tapering of the excitations.

### 3.1. Aperiodic Arrays

For the PS, the results are analyzed in terms of BW and SLL by accounting for four different parameter settings. In particular, each desired pencil beam pattern $F_{d}\left(u\right)$ is characterized either by the half-power beamwidth (HPBW), obtained by setting b = 3 in (3), or by the first-null beamwidth (FNBW), obtained by setting b = 100 in (3). Moreover, two pencil beams are considered: one having BW = 1${}^{\circ }$ and a narrower one having BW = 0.1${}^{\circ }$. The optimal positions are then evaluated according to the Gaussian approach described in Section 2.1 by using (9) in conjunction with (8).

**Figure 3:**
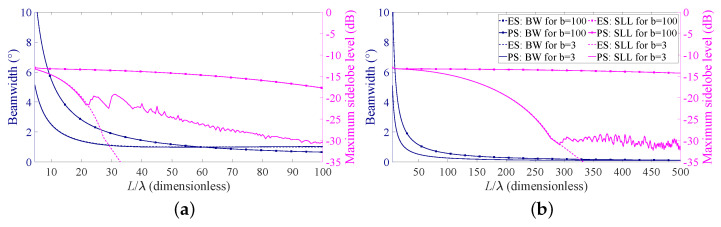
Beamwidth (BW) and sidelobe levels (SLL) as a function of the array aperture. Solid lines refer to PS, dashed lines refer to ES, lines with markers refer to the case b = 100, and lines without markers refer to the case b = 3: (**a**) desired BW = 1${}^{\circ }$ and (**b**) desired BW = 0.1${}^{\circ }$.

The obtained results as a function of the array aperture are reported in Figure 3, where the solid lines refer to the case BW = 1${}^{\circ }$ (Figure 3a) and BW = 0.1${}^{\circ }$ (Figure 3b). As expected, a narrower beam requires a longer array. Thus, in Figure 3b, the analysis is extended to L/λ=500. Moreover, as it can be seen from the figures, in all the cases, the performance improves when the source length is increased and the desired BW is not immediately reached. This is consistent with the well-known relation between BW and antenna length [40]. Furthermore, the obtained SLL is considerably lower when the desired pencil beam is specified in terms of HPBW (lines without markers). Interestingly, in all the examined cases, a sort of minimum attainable SLL seems to exist, which cannot be appreciably decreased by a further increase in the array length. In this regard, it is worth recalling that, in the PS, only the element positions are controlled whereas the element excitations are kept constant. This result may be also consistent with the −13.46 dB limit for the maximum of the first minor lobe of LAAs with uniform spacing and uniform amplitude [40]. It will be shown in the next section, but might also be noticed from Figure 3, that this limit does not seem to exist when amplitude tapering is applied (i.e., in the ES problem).

### 3.2. Periodic Arrays

The results provided by the Gaussian approach for the ES of periodic arrays are now analyzed for the same cases discussed in the previous section by focusing on the interelement distance d=λ/2. This value represents the most common practical choice [40], since the occurrence of grating lobes in the pattern is avoided and the coupling effects are usually assumed negligible. The optimal excitations are evaluated by (10).

The achieved results, still as a function of the array aperture, are illustrated in Figure 3, where the dashed lines identify the obtained beamwidth and SLL for a desired BW = 1${}^{\circ }$ (Figure 3a) and desired BW = 0.1${}^{\circ }$ (Figure 3b). Additionally, in these cases, the performance improves as the source length is increased and the achieved SLL is considerably lower when the desired pencil beam is specified in terms of the HPBW (lines without markers). It is interesting to further observe that, for b = 100, the SLLs derived using the PS and the ES are substantially overlapped, while for b = 3, the ES seems preferable to the PS. In addition, in this latter case, the decrease in the maximum SLL seems not to have a limit, as it was for the PS. This suggests that the SLL can be better controlled when the element excitations can be properly modified. In other words, the ES allows for better control on the maximum SLL compared to the PS.

## 4. Parametric Analysis

As previously stated in the Introduction, the nature of the degrees of freedom provided by periodic and aperiodic arrays is different. Hence, one may expect to not obtain the same performance with the same number of elements. This is however only partially confirmed by the comparison of the SLL curves in Figure 3. More precisely, the magenta dashed line (referring to the ES), after an initial overlapping, becomes considerably lower than the magenta solid line (referring to PS) in both Figure 3a,b. This is, however, true only when the HPBW is considered (lines without markers). Instead, when the FNBW is considered (lines with markers), the curves are overlapped, indicating that the maximum SLLs are substantially the same.

**Figure 4:**
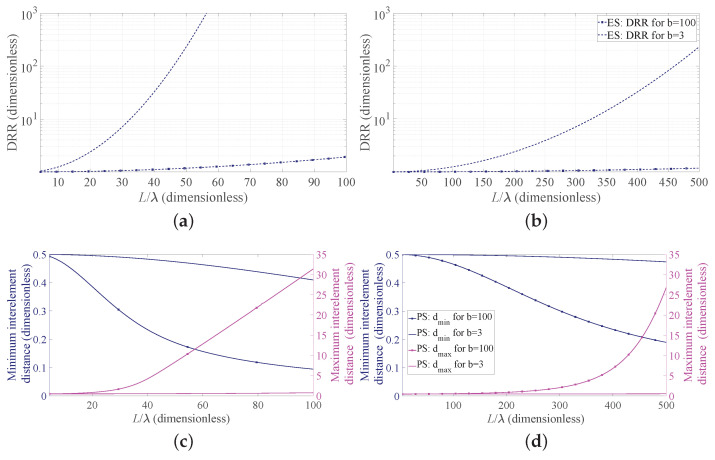
Dynamic range ratio (DRR) and miminum/maximum interelement distance as a function of the array aperture. Lines with markers refer to the case b = 100, and lines without markers refer to the case b = 3: (**a**,**c**) BW = 1${}^{\circ }$, (**b**,**d**) BW = 0.1${}^{\circ }$.

Now, to deepen the discussion regarding the implementation aspects, Figure 4 reports, for the four considered cases, the DRR obtained adopting the ES (Figure 4a,b) and the minimum and maximum interelement spacings normalized to λ obtained using the PS (Figure 4c,d). Note that, in the first two subfigures, the PS results are not reported since the DRR is necessarily equal to one. As it can be seen from these subfigures, when the FNBW is considered (lines with markers), the DRR for both BW values is only slightly larger than unity. This is due to the very narrow desired beam, which provides a flat continuous source a(z) and makes the results of ES essentially identical to those provided by the PS approach. The two second subfigures (Figure 4c,d) reveal an opposite trend. In particular, when the standard deviation σ of the desired pattern is specified in terms of HPBW (b = 3), the maximum and minimum spacings have a small variation as a function of the array normalized length. On the contrary, when the FNBW is used to derive the standard deviation that specifies the desired pattern (b = 100), the obtained spacings vary with the array length. Additionally, these aspects are consistent with the inverse relation between the widths of the desired pattern and that of the corresponding continuous source, represented in Figure 1, and the placement strategy of the Gaussian approach for the PS, represented in Figure 2a. Note that, in Figure 4c,d, the ES results are not present, since the interelement distance is necessarily fixed.

**Figure 5:**
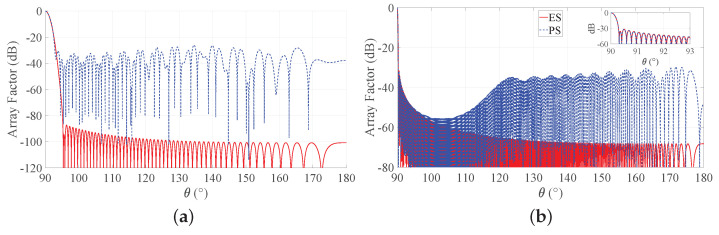
Obtained array factor with b = 3. Blue dashed lines refers to PS, and red solid lines refer to ES: (**a**) desired BW = 1${}^{\circ }$ and L/λ = 60, (**b**) desired BW = 0.1${}^{\circ }$ and L/λ = 300. The inset in the second subfigure reports a zoom of the main beam and of the first sidelobes.

**Figure 6:**
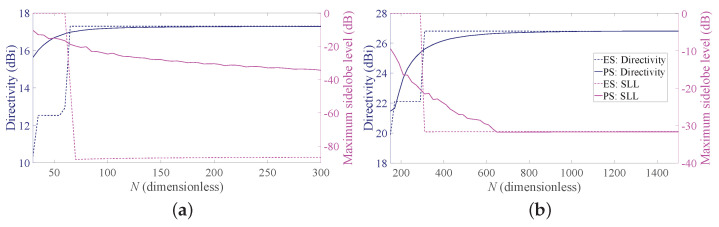
Obtained directivity and SLL as a function of the number of elements. Solid lines refer to PS, and dashed lines refer to ES: (**a**) desired BW = 1${}^{\circ }$, b = 3, L/λ=60, (**b**) desired BW = 0.1${}^{\circ }$, b = 3, L/λ=300.

**Figure 7:**
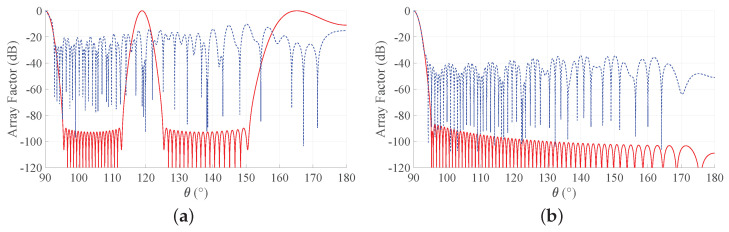

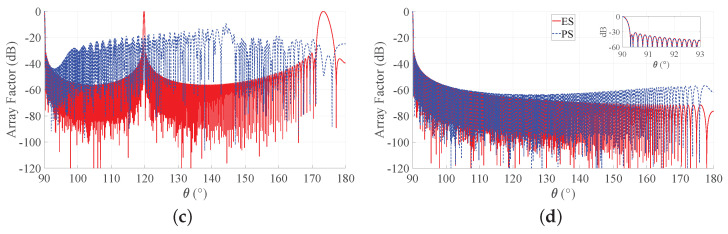
Array factors of the Gaussian approach obtained with b = 3. Blue dashed lines refer to PS, and red solid lines refer to ES: (**a**) desired BW = 1${}^{\circ }$, L/λ=60,N=30, (**b**) desired BW = 1${}^{\circ }$, L/λ=60,N=300, (**c**) desired BW = 0.1${}^{\circ }$, L/λ=300,N=150, and (**d**) desired BW = 0.1${}^{\circ }$, L/λ=300,N=1500. The inset in the last subfigure reports a zoom of the main beam and of the first sidelobes.

To provide a more complete view of the synthesized patterns, Figure 5 compares the array factors of the ES and PS approaches for b = 3 for the two considered BWs. These curves show that the main beam is very well approximated and that the derived SLLs are quite acceptable. With reference to this set of results, it is worth remarking that one of the main advantages of non-periodic arrays is the possibility of reducing the number of elements without introducing grating lobes in the array factors. Hence, a non-periodic array with uniform amplitude distribution may achieve satisfactory performance with a lower number of elements with respect to a periodic array having the same length. Moreover, the DRR of periodic arrays is often very high, as it can be seen from Figure 4. By consequence, it may be desirable to investigate the performance of the Gaussian approach for both PS and ES when the number of elements varies and the array length is fixed. To this aim, Figure 6 shows the directivity and the maximum SLL as a function of the number of elements for an array with L/λ=60 and for a longer one, characterized by L/λ=300. One may immediately note that, when the number of elements increases, the interelement distances decrease. This holds for both periodic and aperiodic arrays. Moreover, in both Figure 6a,b, the low directivity and the 0 dB sidelobes for low *N* values in the ES curves are due to the presence of the grating lobes, which occur since the interelement distance is larger than λ. When, instead, the number of elements increases, the spacing decreases and the performance of ES and PS become similar in terms of directivity, although the sidelobes remain lower in the ES. These aspects can be better appreciated by observing Figure 7, where the array factors derived in the different cases are plotted.

### First-Step Design

Moving from the performed numerical investigations and the corresponding discussion, this subsection describes a procedure to enable an antenna developer to quickly identify the preferable synthesis technique. This procedure exploits the previously presented curves, which are hence treated as design curves. In this regard, it is worth noticing that they have been obtained in a very low computational time, although each of them requires the solution of many synthesis problems. This implies that, if some further curves are required because of their unavailability from the already derived ones, they can be rapidly generated for the required application.

The proposed procedure is explained by moving from a possible synthesis problem, identified by a set of design specifications.

**Statement.** Design a linear broadside array of length L/λ≤100, with HPBW = 1${}^{\circ }$ and maximum SLL not exceeding −20 dB.

**Procedure.** Follow the following steps.

**Figure 8:**
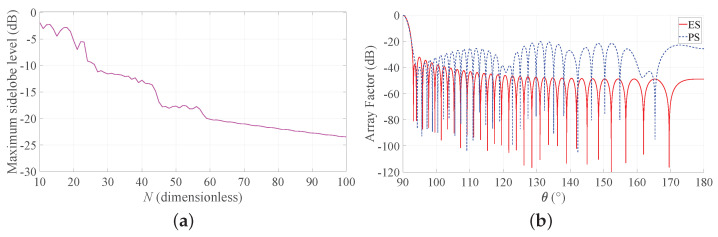
Application example. (**a**) SLL as a function of the number of elements for PS with desired BW = 1${}^{\circ }$, b = 3, L/λ=35. (**b**) The red solid line represents the array factor obtained by ES, and the blue dashed line represents the array factor obtained by PS.

- *Step 1:* Use Figure 3a for the BW requirement. The design curves suggest that an array with L/λ=30 is sufficient to satisfy the requirement in terms of HPBW for both ES and PS. Go to step 2.
- *Step 2:* Use again Figure 3a but now for the SLL requirement. The design curves show that the maximum SLL is considerably lower than the required threshold if ES is adopted, but slightly exceeds the threshold if PS is used. Therefore, if ES is suitable for the specific application, go to Step 3; otherwise, go to Step 4.
- *Step 3:* Use Figure 4a for b = 3 and L/λ=30. The identified point reveals that the DRR of the excitations is approximately 7. If this is acceptable, go to Step 6; otherwise, PS must be adopted. Go to Step 4.
- *Step 4:* Use Figure 3a for the PS with L/λ=30. The relative curve shows that the maximum SLL is slightly higher than the required −20 dB, but the SLL constraint and the BW one are both satisfied when L/λ=35. Go to Step 5.
- *Step 5:* Minimize the number *N* of elements for the PS with BW = 1${}^{\circ }$, b = 3 and L/λ=35 by plotting the required curve in Figure 8a, which as outlined at the beginning of this subsection, requires a low computational time (just 16 ms of CPU time using a commercial personal laptop in this case). This novel curve suggests that N=60 elements allows one to meet the SLL requirement. Go to Step 7.
- *Step 6:* Use (10) in conjunction with (3) to obtain the excitations $a_{n}$, which, using (1), give the array factor in Figure 8b represented by the red solid line. This is the end of the procedure when ES is selected.
- *Step 7:* Use (9) in conjunction with (3) and (8) to obtain the positions $z_{n}$, which, using (1), give the array factor in Figure 8b represented by the blue dashed line. This is the end of the procedure when PS is selected.

As one may immediately observe, the developed procedure enables quick identification of the most suitable approach by adopting design curves that can be rapidly derived. Further adjustments may be subsequently inserted by the antenna designer, but the main choice between ES and PS, given the design specifications, together with the array aperture and the number of elements can be established without performing a large number of preliminary simulations.

## 5. Conclusions

An overview combined with a parametric analysis of the recently developed Gaussian approach has been proposed for both periodic and aperiodic LAAs. The position and excitation synthesis problems have been addressed by comparing the SLL and BW derived, accounting for equal aperture and equal element constraints. This strategy has put into evidence the sensitivity of the solution to the BW specifications, revealing that more satisfactory results are achieved when the main lobe is described in terms of HPBW instead of FNBW. Further investigations have shown that the DRR remains close to unity also when the ES approach is adopted and when very narrow beams are considered. This represents an intrinsic characteristic of the approach and is due to the choice of a Gaussian approximation of the desired beam, a choice that has an influence also on the DRR of the excitations when the ES approach is selected and on the placement strategy and, in particular, on the interelement distances when, instead, the PS approach is preferred. Some limitations might hence arise when very specific main lobe shapes are required, such as in the presence of a desired cosecant beam, which would not be properly fit by a Gaussian function. However, these situations are not common, since, in most cases, the required main lobe is symmetric with respect to the desired direction. The numerical results have in fact shown that the Gaussian approach provides, with conceptual simplicity, a very accurate approximation of the shape of the main beam for both synthesis strategies. These considerations have been exploited to derive a first-step design procedure to enable an antenna engineer to quickly identify the most suitable approach to deal with a given synthesis problem. The main benefit of this procedure may be the reduction in the number of simulation tests and hence of the time required by the design phase.

Current research efforts are devoted to an extension of the analysis to further array geometries and to the derivation of a method to account for mutual coupling effects when the interelement distance becomes lower than half wavelength. 

## Figures and Tables

**Table 1:** PS: performance comparison between different algorithms.

BW	L/λ	*N*	Maximum SLL (dB)						
			[49]	[49]	[43]	[50]	[41]	[46]	Proposed
7.8${}^{\circ }$	4.3	10	−17.09	−17.40	−18.27	–	–	–	−18.36
2.1${}^{\circ }$	16.3	32	–	–	−17.53	–	–	–	−18.10
2.1${}^{\circ }$	9.0	31	–	–	–	−16.88	−18.75	–	−18.89
3.9${}^{\circ }$	9.7	24	–	–	–	–	–	−19.82	−19.71

**Table 2:** ES: performance comparison between different algorithms.

BW	L/λ	*N*	Obtained BW		Maximum SLL (dB)		DRR		SL Power (%)	
			[51]	Proposed	[51]	Proposed	[51]	Proposed	[51]	Proposed
5${}^{\circ }$	20	41	5${}^{\circ }$	5.7${}^{\circ }$	−13.47	−14.27	7.93	1.18	51.54	7.76
5${}^{\circ }$	30	61	5${}^{\circ }$	4.8${}^{\circ }$	−27.01	−21.51	4.16	2.85	5.19	1.47
